# Robot-assisted rehabilitation of people with breast cancer developing upper limb lymphedema: protocol of a randomized controlled trial with a 6-month follow‑up

**DOI:** 10.1186/s13063-023-07778-z

**Published:** 2023-11-15

**Authors:** Federico Arippa, Andrea Scribante, Barbara Rocca, Marco Monticone

**Affiliations:** 1 Department of Medical Sciences and Public Health, University of Cagliari, Cagliari, Italy; 2 Department of Mechanical, Chemical and Materials Engineering, University of Cagliari, Cagliari, Italy; 3 Department of Clinical, Surgical, Diagnostic and Pediatric Sciences, University of Pavia, Pavia, Italy; 4 Department of Clinical Psychology, International Institute of Behavioral Medicines, Sevilla, Spain

**Keywords:** Lymphedema, Rehabilitation, Disability, Machine, Jawbone

## Abstract

Upper limb lymphedema (ULLy) is an external (and/or internal) manifestation of lymphatic system insufficiency and deranged lymph transport for more than 3 months and frequently affects people as a consequence of breast cancer (BC). ULLy is often underestimated despite diminished motor skills, mood, and cognitive-behavioral complaints negatively condition the health-related quality of life (HRQoL) of persons. BC can also metastasize to the jawbone, further impacting on the HRQoL. In time, the implementation of robot-assisted rehabilitation (RR) for neurological diseases has grown to improve HRQoL and pain. This study aims to evaluate the effectiveness of a RR program in the treatment of individuals who develop an ULLy; as a further analysis, the study will assess the effectiveness of the same program in people with jawbone metastases from BC who will also develop ULLy. A randomized, parallel-group superiority-controlled trial will be conducted. 44 participants will be randomly allocated to either the experimental (receiving a RR program) or the control group (regular rehabilitation). Both groups will follow individual-based programs three times a week for 10 weeks. The main outcome measure will be the Lymphedema Quality of Life Questionnaire. Secondary outcomes will be a pain intensity numerical rating scale and the Cranio-Facial Pain Disability Inventory. Evaluations are before and after training and 6 months later. Findings may provide evidence on the effectiveness of a RR program on inducing improvements in the HRQoL and pain of individuals with ULLy due to BC. People with ULLy and jawbone metastases from BC are expected for similar or higher improvements as per the same comparisons above. This trial might contribute towards defining guidelines for good clinical rehabilitation routines and might be used as a basis for health authorities’ endorsements.

**Trial registration** OSF REGISTRIES, osf-registrations-jz7ax-v1. Registered on 26 June 2023.

**Supplementary Information:**

The online version contains supplementary material available at 10.1186/s13063-023-07778-z.

## Introduction

Upper limb lymphedema (ULLy) is an external (and/or internal) manifestation of lymphatic system insufficiency and deranged lymph transport enduring for more than 3 months [1]. 

ULLy frequently affects people as a consequence of breast cancer (BC), being one of the most feared morbidities of this tumor [2].

ULLy is often underestimated despite diminished motor skills, mood, and cognitive-behavioral complaints negatively condition the health-related quality of life (HRQoL) of cancer persons with BC [2–7]. The breast is the more prevalent source for metastatic tumors to the jawbones in women (41%), further contributing to a decrease in the HrQoL of individuals affected by cancer [8, 9].

A rehabilitative approach based on manual therapy, compression bandages along with physical exercises is a common treatment for people with ULLy and usually involves manual lymphatic drainage massage along with elastocompressive therapy, mobilization, muscle strengthening, and stretching of the limb [10, 11].

Robot-assisted rehabilitation (RR) is a rapidly expanding field within human–robot interaction, focused on harnessing the capabilities of robots to assist and enhance human skills during task-centered interactions [12]. RR systems have a wide range of applications, including physical assistance in post-stroke rehabilitation and robotic prosthetics [12, 13] as well as cognitive training for individuals with dementia and Alzheimer’s disease [14, 15]. This interdisciplinary field brings together expertise from various domains and, unlike conventional assistive robotic systems that primarily aim to complete tasks on behalf of users, RR emphasizes training enhancing user skills through interactive experiences. Such instruments maximize the effectiveness and efficiency of training by providing personalized-targeted assistance thus creating an environment that motivates users to perform specific tasks and achieve their training goals [16]. Recent advancements in technologies have facilitated the integration of robots as assistive systems across diverse domains, including also healthcare and physical rehabilitation, mostly focusing on neurorehabilitation in stroke and neurodegenerative diseases. For instance, the iCONE robotic system [17] emerges as an innovative solution for upper limb rehabilitation. By leveraging the principles of RR, the iCONE system enhances the effectiveness and efficiency of therapy delivery. It combines cutting-edge technology, interactive exergames, real-time feedback, and cloud connectivity to provide intensive, engaging, and user-friendly therapy for individuals with upper limb impairments.

We hypothesize that individuals with ULLy due to BC and who undergo RR will experience significant improvements in HRQoL and reduced pain levels similar to or higher than those in the control group based on regular rehabilitation. Specifically, we expect that the RR group will demonstrate higher scores in LYMQoL assessments, and report lower pain scores. We also hypothesize that people with ULLy and who develop jawbone metastases from BC will experience similar or higher improvements as per the same outcome measures as above than those in the control group (an additional self-administered outcome measure as for the assessment of craniofacial disability will be additionally added, as outlined in the Methods).

## Methods

This protocol adheres to the requirements of the Standard Protocol Items: Recommendations for Intervention Trials (SPIRIT) guidelines.

### Trial design and study setting

A single-center, double-blind, two-arm, parallel-group, superiority, randomized controlled trial (RCT) including people with ULLy with 6 months follow-up will be conducted in a secondary care rehabilitation hospital.

### Eligibility criteria

The inclusion criteria will be stable secondary iatrogenic lymphedema with uni- or bilateral swelling of the upper limbs due to BC; age > 18 years; and fluency (i.e., ability to read and write) in Italian. Exclusion criteria will be comorbidities potentially provoking lymphedema (e.g., lipedema, obesity, heart/kidney failure); ongoing chemotherapy/radiotherapy; acute infection (e.g., lymphangitis); other active vascular diseases (e.g., thrombosis); open wounds in the affected limb; mental health/psychiatric deficits (Mini-Mental State Examination score < 24); and refusal to participate.

Inclusion and exclusion criteria will be ruled out based on case histories and routing imaging (e.g. MRI scan, CT scan, ultrasonography, or radioisotope-based lymphoscintigraphy). Any people taking advantage of workers’ compensation or who had formerly undergone regular rehabilitation as ULLy will be excluded.

The presence or absence of jawbone metastases as a consequence of a BC will not influence the inclusion criteria.

### Participants

Between October 2023 and December 2024, persons referring to a secondary care rehabilitation hospital, following an evaluation by two physiatrists coordinated by the principal investigator (PI), will be recruited at the point of care in respect of the inclusion criteria, receiving materials included in a booklet about the study. Participants will be required to declare their consent to participate in the study, and they will be assigned to one of the two treatment programs by the physiatrists. A list of treatment codes and an automatic assignment system to conceal the allocation will be generated and stored through MATLAB and the allocation will follow a permuted-block randomization procedure with a 1:1 rate (an identification number will be assigned to each participant and listed in the dataset; treatment allocation will be automatically generated and concealed by a randomization software). The random sequence will be then converted by the physiatrist in the list of participants assigned to each arm and provided to the physiotherapists and psychologist who will perform the treatment. In order to partially avoid a selection bias, physiatrists will strictly follow the inclusion and exclusion criteria and they will only perform the first evaluation of the participants. The PI will remain blind to the allocation. Personal and occupational variables will be collected for enrolled people.

The PI and whoever will carry out the analysis of the data will be blinded to treatment allocation. The physiotherapists, the psychologist, and the participants will not be blinded.

Research assistants will give additional information to participants, with the latter required to assert their compliance to whichever intervention they are randomly assigned to, and to be present at the follow-up evaluation.

In order to partially limit expectations and to lower crossover troubles, participants will not be told of the trial’s hypothesis but only informed the research was planned to evaluate two interventions as for the rehabilitation of ULLy due to BC whose role had not yet been established.

### Intervention description

The interventions will be performed by two physiotherapists with equal experience (i.e., 10 to 15 years experience in lymphedema rehabilitation). Before the study begins, they will receive adequate training from the physiatrists, each one responsible for one of the two arms of the trial. Following the Italian National Health System protocols on physiotherapy sessions, 30 physical training sessions of 60 min three times per week for 10 weeks will be programmed. At the end of the treatment, participants will be also asked to rate the effectiveness of the treatment using the 5-point Likert Global Perceived Effect (GPE) scale (1 = helped a lot, 2 = helped, 3 = helped only a little, 4 = did not help, 5 = made things worse) [18]. During the program intervention, no other treatment will be performed (e.g., physical modalities, drug therapies for lymphedema), while family doctors will be asked to avoid referring participants to other visits and/or other treatments. Using a specific form, the individuals will be asked to report any serious symptoms they might have during the trial. Participants will be inquired during each session and at the follow-up about their satisfaction with the program intervention; any reasons for skipping the program, seeking additional care, or taking medications to control pain will be discussed. In case of a condition that precludes further participation in the trial or the loss of eligibility (e.g., serious adverse events or other complications; persons with poor compliance that are unable to complete the entire set of exercises; people unable to continue with the clinical trial and request the investigator to withdraw), the participant will be discontinued from trial participation with full disclosure of the reasons and the principal investigator could be informed.

### Robot-assisted Rehabilitation Program (RRP)

The RRP will include robot-assisted exercises for upper-limb muscle isotonic and isometric strengthening (i.e., rotator cuff, thoraco-appendicular, scapulo-humeral, elbow, wrist, and hand muscles) and assisted mobilization of the shoulder (scapula-humeral) and the elbow, along with regular rehabilitation.

In particular, a planar robotic system (iCONE, Heaxel srl, Milan, Italy), already used in previous research on stroke population [17], will be used. This medical device for robot-assisted upper limb rehabilitation incorporates interactive exergames that require coordinated shoulder and elbow movements to perform planar reaching tasks displayed on a monitor. The device administers protocols based on intensive therapeutic exercise repetitions, guiding participants to specific points on the display, so that users receive real-time visual feedback on their position and movement accuracy.

The RRP will last for 60 h in total as a result of the presence of the robot-assisted exercises added to regular rehabilitation (described below).

### Regular rehabilitation

It will include simultaneously: (i) manual lymphatic drainage massage; (ii) elastocompressive therapy; and (iii) exercises for the head, neck, and upper-limb muscle isotonic and isometric strengthening (i.e., rotator cuff, thoraco-appendicular, scapulo-humeral, elbow, wrist and hand muscles), regional stretching (i.e., shoulder girdle and upper limb muscles), and assisted mobilization of the temporo-mandibular, shoulder, elbow, wrist, and hand joints [19].

The general program interventions will last for 30 h in total.

### Outcomes

#### Primary outcome measure

The primary outcome measure will be the change from the baseline of the Function domain in the Lymphedema Quality of Life (LYMQOL) questionnaire [20, 21] at 10 weeks and after 6 months. The LYMQOL is a self-administered questionnaire consisting of 21 items. The first 20 items assess the impact of lymphedema on Health-Related Quality of Life (HRQoL) and are categorized into four domains: Function (items #1–3), Appearance (items #4–8), Symptoms (items #9–14), and Mood (items #15–20). Each item is rated on a 4-point scale, ranging from 1 (not at all) to 4 (a lot), with higher scores indicating lower HRQoL.

The scores of all items within that domain are summed and divided by the total number of questions answered, in order to calculate the total score for each domain. Additionally, item #21 assesses the overall HRQoL, using an 11-point numerical scale ranging from 0 (poor HRQoL) to 10 (excellent HRQoL). The four summary scores related to each domain will be computed according to the guidelines provided by Keeley and colleagues [20].

#### Secondary outcome measure

The secondary outcome measures include the following:

- Change from baseline of the Appearance, Symptoms, and Mood domains of the LYMQOL at 10 weeks and 6 months.
- Change from baseline of the Numerical Rating Scale (NRS) [22] at 10 weeks and 6 months. The NRS is a measure of pain intensity, using an 11-point numerical rating scale ranging from 0 (no pain) to 10 (the worst imaginable pain).
- Change from baseline of the Craniofacial Pain Disability Inventory (CFPDI-I). This inventory consists of 21 items, and participants provide their answers using a 4-point Likert scale ranging from 1 (no problem) to 4 (maximum problem). The inventory has two factors: Pain and disability (14 items) and Jaw functional status (7 items). The scores of the responses are summed, with higher scores indicating a greater problem [23, 24]. This outcome measure will be administered to people with jawbone metastases due to BC only.

All of these outcome measures will be administered at three time points: before the rehabilitation program, at the end of the 10-week rehabilitation program, and at a follow-up assessment 6 months after the completion of the rehabilitation program.

Further, the outcome measures are furnished by secretarial staff (i.e., a physiotherapist with a master’s degree, who will not participate in any part of the study) who will check them and return any unfinished sections to participants. At follow-up, the people involved in the study will be contacted (face to face or, if not possible, via telephone) individually by the same personnel to warrant the outcome measures are accurately completed.

### Participant timeline

**Fig. 1:**
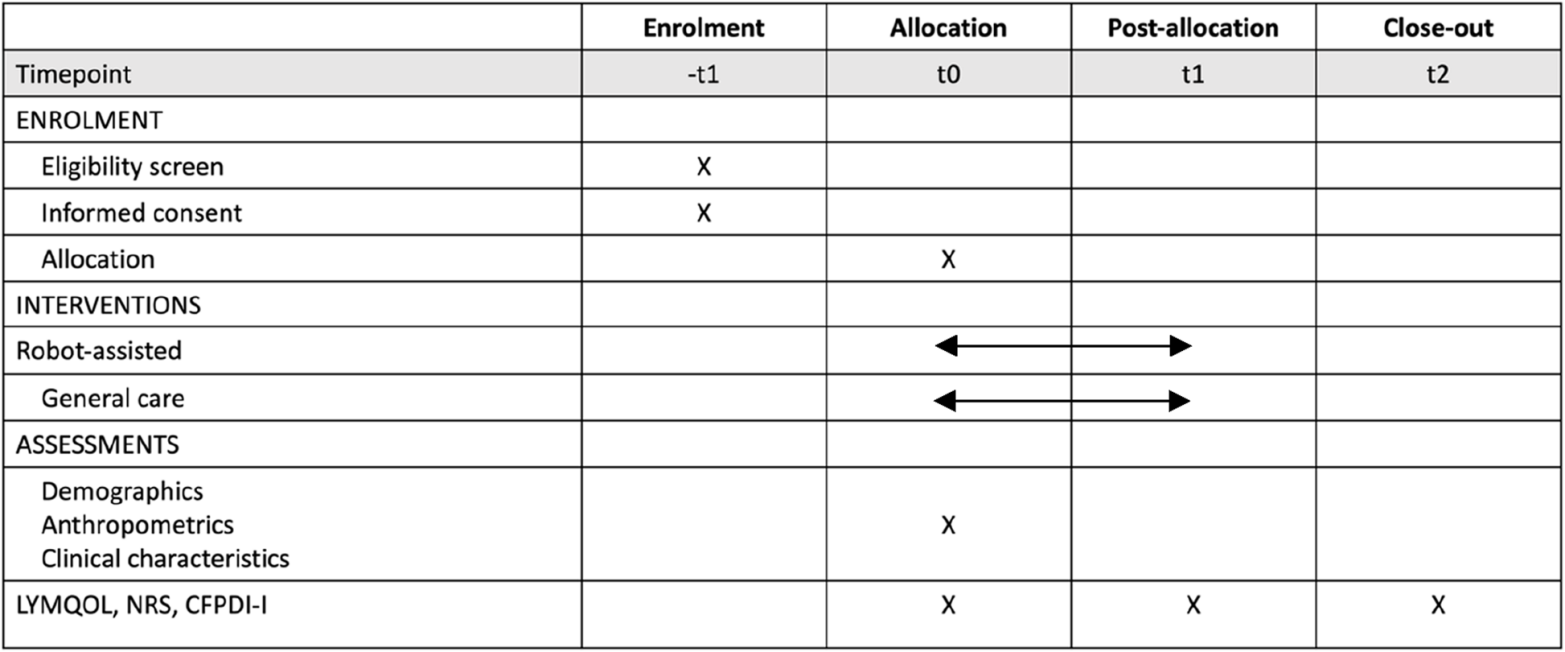
Schedule of enrolment, interventions, and assessments. LYMQOL, Lymphedema Quality of Life; NRS, Numerical Rating Scale; CFPDI-I, Craniofacial Pain Disability Inventory

The participant timeline is shown in Fig. 1.

### Data collection and management

The data collection and analysis processes will be conducted by the PI and biostatistician, respectively. Compliance with the European Regulation on the Protection of Personal Data (GDPR 2016/679) will be ensured, specifically with regard to the principles applicable to the processing of personal data (Article 5), lawfulness of processing (Article 6), and conditions for consent (Article 7).

All collected data will be used solely for scientific research purposes and will be securely stored to prevent unauthorized access. Measures will be taken to remove any information that could potentially identify the participants, ensuring their anonymity. Each participant will be assigned a unique identification number, which will be used in the dataset. The responsibility for keeping the data will lie with the principal investigator.

### Sample size and statistical analysis

As there is no defined minimally important change (MIC) for breast lymphedema tools [25] that represent the primary outcome of this study, the estimated sample size is made on a medium effect size (*η*^2^ = 0.06). With a type I error of 0.05 and a power of 90%, the calculated sample size is a minimum of 36 participants. Considering a dropout rate of 20%, a total of 44 participants will be recruited.

To analyze measures with non-normal distribution, the Mann–Whitney *U* test will be employed to examine the differences between the two groups. For assessing mean changes in primary and secondary outcomes between the intervention and control groups over time on an intention-to-treat basis, linear mixed-effects models for repeated measures (*p* < 0.05) will be utilized [26, 27]. The null hypothesis will be rejected for *p*-values greater than 0.05 using a two-tailed test. If needed, additional analyses including subgroup and adjusted analyses, will be completed. Subgroup analyses, stratified for age, gender, and disease severity, may be conducted to investigate potential differences and interactions within the study population. Adjusted analyses may also be performed to account for potential confounding factors.

All statistical analyses will be performed using SPSS 21.0 (IBM SPSS Statistics, Armonk, USA) software. Results for normally distributed continuous variables will be expressed as mean ± standard deviation, while continuous variables with non-normal distribution will be presented as median values and interquartile range. Demographic and baseline characteristics will be summarized using descriptive statistics, and categorical variables will be reported as absolute numbers and percentages.

No interim analyses or formal stopping rules will be performed due to the small sample size considered and the relatively short duration of the trial. Indeed, the considered sample lacks the statistical power needed for interim assessments, increasing the risk of type I errors and data-driven bias. Moreover, considering the short duration, formal stopping rules are not necessary.

### Discontinuation criteria and treatment

- Participants who experience serious adverse events or complications, as determined by the investigator, will be discontinued from the trial and provided with appropriate treatment.
- Participants who demonstrate poor compliance and are unable to complete the full set of exercises, thereby impacting the study results, will be discontinued.
- Participants who, for any reason, express unwillingness or inability to continue with the clinical trial and request withdrawal will be discontinued from the study.

For cases of discontinuation, noncompliance, or loss to follow-up, missing data analysis will be conducted to examine patterns in missing variables. Specifically, we will assess whether the missing values are likely to be missing at random (MAR), missing completely at random (MCAR), or missing not at random (MNAR). If the data is MCAR or MAR, we will employ multiple imputation techniques and present the results with and without the imputed data. In the case of MNAR data, missing values will not be replaced, and case analysis will be conducted when available. In linear mixed-effects models for repeated measures, unbiased estimation of the treatment effect will be adjusted for time-varying confounders.

To compare participants' characteristics at baseline between groups, independent samples t-tests will be used for age, pain duration, body mass index, and outcome measures. Pearson chi-squared test will be utilized for categorical variables such as gender, occupation, and type of exercise. Linear mixed model analyses for repeated measures, with a significance level of 5%, will be conducted for each measure, with group and time included as fixed effects and the outcome measure as the dependent variable. The time by group interaction term will also be evaluated. Between-group differences at the end of the program and follow-up will be assessed for the primary outcome and other variables.

### Auditing

Auditing sessions will be conducted by physiatrists at the conclusion of the treatment and follow-up periods. The PI will not be involved in the auditing process, ensuring independent analysis.

### Dissemination plans

The findings and scientific insights obtained from this trial will be published in national and international scientific journals with a peer-review system. The intellectual property rights of the data will belong to the academic departments which the investigators belong to. They will have the freedom to publish the data, present them at conferences, and share them through various scientific and public platforms deemed appropriate for the dissemination of the acquired knowledge.

## Discussion

ULLy is a frequent complication of BC and causes disability and pain in the people affected BC [2–7]. Further, these individuals experience difficulties during activities of daily living and, when adult ages are involved, also work difficulties may take place [28, 29]. Economic and social costs for both people and society are also present [30].

Although it is already known that multidisciplinary programs that include robot-assisted and exercise therapy can lead to significant and lasting changes in physical impairment, pain, and quality of life of persons with other musculoskeletal and neurological disabilities [12, 13, 15–17, 31, 32], it is still questioned if similar programs may have the same results on people with ULLy. The planned trial is expected to innovatively fill the gap of knowledge within this field which may have important clinical and research impact. In more detail, this trial is expected to provide new data on the effectiveness of a RRP on inducing clinically significant improvements in the HrQoL and pain of people with ULLy due to BC (including also those with jawbone metastases), and that these results would be maintained in the medium term, compared to regular rehabilitation. Further, the data that will be collected are expected to influence extra developments of RR, an ever-increasing biomedical field which may be of support to people with BC. Furthermore, the data which will be gathered might also favor a deeper understanding as concerns the relationships in persons with jawbone metastases between two outcome measures (i.e., the LIMQOL, as for the upper limb region, and the CF-PDI as for the head area [20, 23]), as previously described.

Definitely, this trial might contribute towards refining guidelines for good clinical practice and might be used as a basis for health authorities’ recommendations.

Some limitations affect the present trial. Firstly, data monitoring committee (DMC) is not provided; however, given the short-term duration of the follow-up (6 months) involving low risks for patients under treatment and without critical safety concerns, mainly evaluating relief of symptoms as outcome, the DMC can be omitted without any risk for participants and the results of the trial. Secondly, there will be contact-time differences between the groups due to the robot-assisted intervention. Third, post-trial care has not been accounted for.

## Trial status

Protocol final version—July 10 2023: recruitment will start on October 2023 and approximately will be completed on December 2024.

In the event of protocol amendments, the sponsor and funder will be notified first. Subsequently, the Principal Investigator will inform the centers. A copy of the revised protocol will be added to the Investigator Site File, and any deviations from the Protocol will be documented. The protocol will be updated in the OSF registries website.

### Supplementary Information

**Additional file 1.**

## Data Availability

The datasets of this study will be not publicly available due to individual privacy rules; only the principal investigator can access the blinded dataset, as well as biostatistics in order to perform data analysis. Anonymized dataset and statistical code will be made available under motivate request at the corresponding author. Only the principal investigator can share the requested data, after the evaluation of the motivation provided by the claimant.

## Abbreviations

BC: Breast cancer
GPE: Global Perceived Effect
HrQOL: Health-related quality of life
LYMQOL: Lymphedema Quality of Life
ULLy: Upper limb lymphedema
NRS: Numerical Rating Scale
CF-PDI: Craniofacial Pain Disability Inventory
MIC: Minimally important change
RRP: Robot-assisted rehabilitation
RCT: Tandomized controlled trial
DMC: Data monitoring committee
MAR: Missing at random
MNAR: Missing not at random
